# Frontiers in Drug Research and Development for Inflammatory Bowel Disease

**DOI:** 10.3389/fphar.2017.00400

**Published:** 2017-06-23

**Authors:** Diego Currò, Daniela Pugliese, Alessandro Armuzzi

**Affiliations:** ^1^Institute of Pharmacology, School of Medicine, Catholic University of the Sacred HeartRome, Italy; ^2^IBD Unit, Internal Medicine and Gastroenterology, Fondazione Policlinico Universitario “A. Gemelli” Presidio Columbus, Catholic University of the Sacred HeartRome, Italy

**Keywords:** inflammatory bowel disease, S1P receptors, integrins, MAdCAM-1, interleukin-23, interleukin-6, Janus kinases, Smad7

## Abstract

Inflammatory bowel disease (IBD) is idiopathic, lifelong, immune-mediated diseases, for which curative therapies are not yet available. In the last 15 years, the introduction of monoclonal antibodies targeting tumor necrosis factor-α, a cytokine playing a key role in bowel inflammation, has revolutionized treatment paradigms for IBD. Despite their proven long-term efficacy, however, many patients do not respond or progressively lose response to these drugs. Major advances of knowledge in immunology and pathophysiology of intestinal inflammatory processes have made possible the identification of new molecular targets for drugs, thus opening several new potential therapeutic opportunities for IBD. The abnormal response of intestinal immunity to unknown antigens leads to the activation of T helper lymphocytes and triggers the inflammatory cascade. Sphingosine 1-phosphate receptor agonists negatively modulate the egress of lymphocytes, inducted by antigen-presenting cells, from secondary lymphoid tissues to intestinal wall. Leukocyte adhesion inhibitors (both anti-integrin and anti-Mucosal Vascular Addressin Cell Adhesion Molecule 1) interfere with the tissue homing processes. Activated T helper lymphocytes increase the levels of pro-inflammatory cytokines, such as interleukin 12, 23, and 6, offering several potential pharmacological interventions. The Janus kinases, intracellular enzymes mediating the transduction of several cytokine signals, are other explored targets for treating immune-mediated diseases. Finally, the impact of modulating Smad7 pathway, which is responsible for the down-regulation of the immunosuppressive cytokine transforming growth factor-β signaling, is currently under investigation. The purpose of this review is to discuss the most promising molecules in late-stage clinical development, with a special emphasis on pharmacological properties.

## Introduction

Inflammatory bowel disease, including UC and CD, are chronic, idiopathic, relapsing inflammatory disorders of the intestine that recognize an unknown etiology and complex and poorly defined pathogenesis. Currently, many drugs are available for the treatment of IBD, conventionally subdivided into two main classes: traditional drugs and biologics. The first class of drugs includes the aminosalicylates (mainly 5-aminosalicylic acid [mesalamine]), corticosteroids, and the immunosuppressive drugs (mainly azathioprine, methotrexate, and cyclosporine). As far as biologics are concerned, several anti-TNF-α monoclonal antibodies (infliximab, adalimumab, certolizumab, and golimumab) and recently monoclonal antibodies direct against α_4_β_7_ integrin (vedolizumab) and subunit p40 of IL-12/IL-23 (ustekinumab) have become available (Deepak and Bruining, 2015; Deepak and Loftus, 2016; Ungaro et al., 2016). The traditional treatment approach for IBD patients is based on a “step-up strategy,” with a progressive escalation to more powerful but potentially more harmful therapies according to disease severity and response to previous drugs (Deepak and Bruining, 2015; Ungaro et al., 2016). At the bottom of this “therapeutic pyramid,” there are drugs better tolerated and effective in patients with mild to moderate IBD, including aminosalicylates, topical corticosteroids and antibiotics. Systemic corticosteroids are positioned on the next step and are prescribed in case of moderate-to-severe disease or non-response to aminosalicylates. Patients developing steroid-dependence or resistance are escalated to traditional immunosuppressive drugs (azathioprine and methotrexate). The most effective and potentially least tolerated drugs, that is biological drugs and cyclosporine (this last reserved for acute severe UC), occupy the top position of the pyramid (Antunes et al., 2014; Deepak and Bruining, 2015; Ungaro et al., 2016). Biological therapies are recommended for patients with moderately-to-severely active disease, who have not responded to, are intolerant to or have medical contraindications for corticosteroids and/or immunosuppressants. However, available monoclonal antibodies showed limited efficacy. Regarding anti TNF-α (on which we have the longest real-life experience), approximately 30% of treated patients do not respond from the beginning and up to 25% per year progressively lose the response (Allez et al., 2010; Ben-Horin and Chowers, 2011). Thus, there is a growing interest in developing new drug therapies based on pathophysiological mechanisms of the intestinal inflammatory process.

In the pathophysiological cascade of IBD, the adaptive immune responses play major roles, in particular those mediated by Th lymphocytes. Current hypothesized pathogenetic mechanism of IBD involves as initial events the alteration of gut microbiota and the impairment of intestinal epithelial barrier function, in genetically predisposed hosts, by environmental factors. The consequent exposure of the intestinal mucosal immune system to altered microbial antigens triggers a dysregulated immune response that results in inflammation of the intestinal wall (Zundler and Neurath, 2015). The microbial antigens would activate the antigen-presenting cells, in particular dendritic cells. The latter migrate to secondary lymphoid tissues, in particular to mesenteric lymph nodes, where they present the antigens to the resident naive T lymphocytes, inducing their differentiation into Th cells, proliferation (clonal expansion) and expression of trafficking surface molecules that target them to the intestinal wall. Subsequently, these specific Th lymphocytes have two possibilities: either become resident memory cells or egressing from lymph nodes reach the intestinal wall, where, once homed, they secrete ILs and other cytokines responsible for intestinal inflammation. Consequently, drug research and development for IBD has followed new directions aimed to synthesize molecules that inhibit some of the pathogenetic steps listed above, including the egress of lymphocytes from the secondary lymphoid organs, their homing in the intestinal tissues or the actions of pro-inflammatory cytokines, or promote the activity of anti-inflammatory cytokines.

## Drugs that Interfere with the Egress of Immune Cells from Lymphoid Tissues

The egress of activated lymphocytes from secondary lymphoid tissues is the first event of the process called cell trafficking, which consists in a series of consecutive steps by which immune cells, primarily lymphocytes, move from secondary lymphoid tissues, such as lymph nodes and gut-associated lymphoid tissue, to peripheral tissues and organs, including the intestinal submucosa and mucosa. S1P plays a relevant role in this process. This mediator belongs to the family of lyso-phospholipids, and is produced by the phosphorylation of sphingosine by the enzymes sphingosine kinase-1 and -2. Sphingosine, in turn, generally derives from the hydrolysis of sphingosine ceramides (*N*-acyl-sphingosines), lipid components of the plasma membrane, by some ceramidases. S1P is an important signaling molecule that plays physiological roles in the processes of cell growth, proliferation and survival, cell migration and chemotaxis, neurogenesis and angiogenesis, heart inotropism and chronotropism, endothelial permeability, and blood vessels integrity and tone (Proia and Hla, 2015; Vogt and Stark, 2017). Moreover, it is also involved in pathophysiological processes, such as inflammation and cancer (Maceyka and Spiegel, 2014; Pyne et al., 2016). S1P is mostly secreted by erythrocytes, vascular and lymphatic endothelial cells, and platelets through a carrier-mediated mechanism (Proia and Hla, 2015). It is the orthosteric ligand of S1P receptors, which are class A G-protein coupled receptors classified in five subtypes (S1P_1_ to S1P_5_ receptors), belonging to the family of lysophospholipid receptors (Chun et al., 2010). S1P_1-3_ receptors are widely expressed in the body organs and tissues, in relation to their abundant expression in endothelial cells (Proia and Hla, 2015), whereas S1P_4-5_ receptors show a more limited expression (S1P_4_ receptors in lymph tissue and lung, and S1P_5_ receptors in brain, skin, and spleen; Vogt and Stark, 2017). S1P_1_ and S1P_4_ are the main S1P receptors expressed in lymphoid tissue (Vogt and Stark, 2017), particularly in lymphocytes and dendritic cells (Karuppuchamy et al., 2017). S1P binding to these receptors, particularly to S1P_1_ receptors, induces lymphocyte migration out from secondary lymphoid tissues along a S1P concentration gradient toward lymph and blood, which have S1P concentrations (0.1 and 1 μM, respectively) higher than that in lymph node interstitial fluid (Proia and Hla, 2015). The high blood S1P concentrations desensitize S1P_1_ receptors by inducing phosphorylation through G protein coupled receptor kinases and consequent receptor internalization, followed by receptor re-sensitization and re-exposure in the plasma membrane. Similarly, S1P receptor agonists (also named “modulators”) induce internalization of membrane S1P_1_ receptors in the secondary lymphoid tissues by the same mechanisms. However, the receptor internalization induced by the continuous exposure to effective concentrations of receptor agonists seems to be followed by receptor ubiquitination and degradation through the proteasome in lymphocytes causing long-lasting receptor downregulation and lymphocyte sequestration in lymphoid tissues (preventing them from reaching the inflammation sites). Fingolimod (FTY720) was the first S1P receptor agonist to be approved for clinical use, currently on the market for the treatment of relapsing remitting MS. It is a prodrug phosphorylated by sphingosine kinase-2; in its phosphorylated form, it is conventionally considered a high affinity agonist for S1P_1_ and S1P_3-5_ receptors, even though it also binds to S1P_2_ receptor with lower affinity (Sobel et al., 2015). In lymphocytes, phosphorylated fingolimod binds mainly to S1P_1_ receptors, inducing the sequestration of these cells in secondary lymphoid tissues and a long-lasting reduction of the lymphocyte count in peripheral blood and tissues. Fingolimod has not been developed clinically for the treatment of IBD, even though many studies show its efficacy in reducing the intestinal inflammation in murine models of IBD (Deguchi et al., 2006; Fujii et al., 2006; Daniel et al., 2007a,b; Fujii et al., 2008; Radi et al., 2011). Three other S1P receptor agonists are being developed for clinical use in IBD, namely ozanimod (RPC1063), amiselimod (MT-1303) and etrasimod (APD-334). All of them have a narrower range of S1P receptor subtype selectivity. **Ozanimod** (**Table 1**) is a S1P_1_/S1P_5_ receptor agonist with potency approximately 27 times higher for S1P_1_ receptors than for S1P_5_ receptors (EC_50_s for S1P_1_ and S1P_5_ receptors: 0.41 and 11 nM, respectively; Scott et al., 2016). Ozanimod induces a dose-dependent and long-lasting reduction of S1P_1_ receptor density in transfected CHO cells, which is almost complete at concentrations slightly >10 nM (Scott et al., 2016). In correlation with this effect, ozanimod also produces a dose-dependent decrease in circulating T lymphocytes in rodents, which is almost completely reversible within 24 h, unlike that induced by fingolimod (Scott et al., 2016). This difference seems to be related to the shorter t_1/2_ of ozanimod compared to fingolimod (approximately 5 h vs. 15–34 h, respectively, in rodents). Ozanimod was also shown to decrease the indices of inflammation in two experimental models of IBD in rodents, that is the colitis produced by 2,4,6-trinitrobenzenesulfonic acid and T cell adoptive transfer (Scott et al., 2016). Ozanimod efficacy and safety in patients with UC were evaluated in the TOUCHSTONE study, a phase 2, multicentre, double blind, randomized and controlled trial (RCT). In total, 197 patients, with moderate-to-severe UC, were randomly assigned to receive ozanimod 0.5 mg or 1.0 mg once daily, or placebo (1:1:1) through week 32. At week 8, clinical remission, defined as full Mayo score (FMS) ≤ 2 with no sub-score >1 (Silverberg et al., 2005), was achieved in a higher percentage of patients treated with ozanimod compared to those receiving placebo (1 mg 16.4%, 0.5 mg 13.8%, and placebo 6.2%, *p* = 0.0482; **Table 2**). At week 8, the clinical response (defined as reduction in FMS of ≥3 points and ≥30%, with a decrease in the rectal bleeding score of ≥1 or a rectal bleeding score ≤1) and the mucosal improvement (Mayo endoscopic sub-score ≤1) were also significantly greater in both ozanimod groups than in the placebo one (Sandborn et al., 2016b). After induction, 103 patients (52.3%), who were in clinical response, continued with the blinded treatments for additional 24 weeks. At week 32, the proportions of patients who maintained a clinical remission were significantly greater in both active groups (1 mg 20.9% and 0.5 mg 26.2%, respectively) compared with the placebo one (6.2%; *p* = 0.0108 and *P* = 0.0002 vs. placebo, respectively; **Table 2**). Patients in both active arms were also more likely to achieve 32-week clinical response and mucosal improvement (Sandborn et al., 2016a). Furthermore, histological remission (defined by a Geboes score grade < 2; Bessissow et al., 2012) was recorded in a higher percentage of patients treated with ozanimod compared to those receiving placebo at both week 8 and week 32 (Sandborn et al., 2016a,b). After the completion of the study, 170 patients (86%) entered the OLE study, receiving ozanimod 1 mg once daily up to week 80. At the time of OLE entry, approximately 34% of patients were in clinical remission (defined as rectal bleeding score of 0 and stool frequency ≤ 1) and 27% showed histological remission. The percentage of patients in clinical remission increased throughout OLE, up to 62% at both week 32 and 44, and 55% at week 80. Patients who entered OLE in histological remission and after receiving active treatment in the first blinded 32 weeks were more likely to achieve clinical remission (almost 90% at OLE week 4 and 8). As far as safety is concerned, ozanimod was well tolerated, with similar incidences of AEs across treatment groups. No AEs of special interest were recorded, but transient asymptomatic increases in serum transaminases were recorded in 3% of patients (Sandborn et al., 2017c). Similar to fingolimod, **amiselimod** is a prodrug converted in a S1P_1_/S1P_5_ agonist through phosphorylation by sphingosine kinases (Sugahara et al., 2017). Amiselimod phosphate is approximately six times more selective for S1P_1_ than for S1P_5_ receptors (EC_50_s for S1P_1_ and S1P_5_ receptors: 0.075 and 0.47 nM, respectively; Sugahara et al., 2017). Amiselimod phosphate also binds to S1P_4_ receptors, but with an apparent affinity approximately 1,630 times lower than that for S1P_1_ receptors (EC_50_ for S1P_4_ receptors: 122.3 nM; Sugahara et al., 2017). Amiselimod is activated more slowly than fingolimod in human cardiomyocytes, and this finding has been related to the more favorable cardiac safety profile of amiselimod with respect to fingolimod (Harada et al., 2017). Bradycardia is a well-known acute unwanted effect of fingolimod, reported as symptomatic in approximately 0.6% of treated patients in clinical phase 3 trials. Thus, the regulatory authorities have prescribed cardiac monitoring for at least 6 h during treatment initiation. Bradycardia, in humans, seems to be mainly attributable to activation of S1P_1_ receptors. Differently from fingolimod, amiselimod did not significantly reduce the heart rate during the first 2 days of treatment, and did not induce any clinically significant bradyarrhythmia in two phase 1 clinical studies (Harada et al., 2017; Sugahara et al., 2017). Amiselimod induces dose-dependent reductions in peripheral lymphocyte counts in humans. With single daily dosing, the reductions reach a plateau after approximately 14 days and are 60–70% of baseline values after 21 days at doses of 0.5–0.75 mg once daily (Sugahara et al., 2017). These findings were confirmed in a second phase 1 clinical study (Harada et al., 2017). The return of lymphocyte counts toward pre-treatment levels after dose interruption is gradual, and reaches 77.1 and 59.8% of baseline values after 49 days in subjects receiving amiselimod 0.4 and 0.8 mg once daily for 28 days, respectively (Harada et al., 2017). The gradual recovery of lymphocyte counts toward baseline levels is attributable to the long t_1/2_ of amiselimod and its phosphorylated metabolite (approximately 430–440 h with respect to 255 h for fingolimod; Harada et al., 2017). **Etrasimod** is a selective modulator of S1P_1_ and S1P_5_ receptors (Buzard et al., 2014). The potency of etrasimod on S1P_1_ receptors, evaluated by a β-arrestin recruitment assay, is four times higher than that on S1P_5_ (EC_50_s for S1P_1_ and S1P_5_ receptors: 6.1 and 24.4 nM, respectively; Buzard et al., 2014). Etrasimod also activates S1P_4_ receptors, but with a potency approximately 24 times lower than that for S1P_1_ receptors (EC_50_ for S1P_4_ receptors: 147 nM; Buzard et al., 2014). In addition, efficacy of etrasimod at S1P_4_ and S1P_5_ receptors is lower than that at S1P_1_ receptors (E_max_s for S1P_4_ and S1P_5_ receptors: 63 and 73%, respectively; Buzard et al., 2014). Etrasimod induces S1P_1_ receptor internalization in CHO cells expressing the receptors with an EC_50_ of 1.88 nM, and lymphopenia with plasmatic EC_50_ values of 0.05–0.1 μM in mice, rats, dogs, and monkeys (Buzard et al., 2014). Etrasimod has a bioavailability ranging from approximately 44% in monkeys to 100% in mice, and its t_1/2_ is included between approximately 6.4 h in monkeys and 29 h in dogs (Buzard et al., 2014). In phase 1 clinical studies, etrasimod was well tolerated in healthy adults after administration of single doses in the range 0.1–3 mg, whereas atrioventricular block was observed in 50% of subjects taking the 5-mg single dose (Peyrin-Biroulet et al., 2017a). The drug induced dose-dependent arterial hypotension and bradycardia both in single dose and in repeated doses (0.7–3 mg once daily for 21 days) studies. Two cases of atrioventricular block were observed in subjects taking etrasimod 2 or 3 mg in the multiple dose study. Etrasimod dose-dependently reduced the peripheral lymphocyte counts, and a steady state effect was obtained with the dose of 2 mg once daily (median reduction of approximately 67%). Lymphocyte counts returned to baseline levels within a week of dosing discontinuation (Lee et al., 2017). Etrasimod decreased the peripheral T cell counts, in particular those of Th and T-naive lymphocytes; counts of T-suppressor and T-effector memory cells were not generally lowered (Lee et al., 2017). The plasma exposure to etrasimod was dose-dependent in the dose range 0.1–5 mg, and the mean terminal t_1/2_ was approximately 31–37 h (Kühbacher et al., 2017). With multiple once daily dosing, a two-fold to three-fold etrasimod plasma accumulation was observed at the end of the 21-day administration period. In the IBD experimental model of severe combined immunodeficiency mice subjected to CD45RB^high^ CD4^+^ T cell adoptive transfer, etrasimod significantly reduced the colonic inflammation indices and weight loss with respect to controls (Kühbacher et al., 2017). Amiselimod and etrasimod are currently under testing in phase 2 trials for IBD, in CD (NCT02447302 and NCT02536404) and UC (NCT02378688 and NCTB02389790) patients, respectively, but preliminary data are not yet available. **KRP-203** is another compound of the same class, previously tested in a phase 2 trial in UC. However, KRP-203, even though showing good safety profile, did not reach the minimal threshold of clinical relevance (Radeke et al., 2016).

**Table 1:** Target processes or pathways of new drugs with evidence for clinical efficacy in patients with ulcerative colitis (UC) or Crohn’s disease (CD).

Target process	Drug modulatory effect on target process	Drug	Drug type	Mechanism of action	Currently proven clinical efficacy in IBD
Egress of immune cells from lymphoid tissues	Inhibition	Ozanimod (RPC1063)	Small molecule	S1P receptor agonist	UC
Immune cell homing	Inhibition	Abrilumab (AMG 181 or MEDI7183)	Biological	Anti-α_4_β_7_ monoclonal antibody	UC
		AJM300	Small molecule	Integrin antagonist (by binding to the α_4_ subunit)	UC
		Etrolizumab (rhuMAb Beta7 or PRO145223)	Biological	Anti-β_7_ subunit monoclonal antibody	UC
		PF-00547659	Biological	Anti-MAdCAM-1 monoclonal antibody	UC
Th17 cell pathway	Inhibition	Risankizumab (BI 655066 or ABBV-066)	Biological	Monoclonal antibody directed toward the p19 subunit of IL-23	CD
		Brazikumab (AMG 139 or MEDI2070)	Biological	Monoclonal antibody directed toward the p19 subunit of IL-23	CD
IL-6 pathway	Inhibition	PF-04236921	Biological	Anti-IL-6 monoclonal antibody	CD
JAK/STAT signaling pathway activated by class I and II cytokines	Inhibition	Tofacitinib (CP-690,550)	Small molecule	Non-selective JAK inhibitor	UC
		Filgotinib (GLPG0634 or GS-6034)	Small molecule	JAK1 inhibitor	CD
TGF-β pathway	Amplification	Mongersen (GED0301)	Antisense oligonucleotide	Smad7 inhibitor	CD


*JAK, Janus kinase; IL, interleukin; MAdCAM-1, Mucosal Vascular Addressin Cell Adhesion Molecule 1; STAT, Signal Transducer and Activator of Transcription; S1P, sphingosine 1-phosphate; TGF, transforming growth factor.*

**Table 2:** Summary of clinical trials for new drugs in clinical development for moderate-to-severe ulcerative colitis.

Drug (route of administration)	Study reference	Study phase	Primary endpoint	Drug dose (No. of patients)	% of patients who achieved the primary endpoint
Ozanimod (p.o)	Sandborn et al., 2016b	2	Induction of clinical remission at week 8, defined as FMS ≤ 2	0.5 mg once daily (65)	13.8
				1 mg once daily (67)	16.4^∗^
				Placebo (65)	6.2
	Sandborn et al., 2016b	2	Maintenance of clinical remission at week 32, defined as FMS ≤ 2	0.5 mg once daily (65)	26.2^∗^
				1 mg once daily (67)	20.9^∗^
				Placebo (67)	6.2
Abrilumab (s.c.)	Sandborn et al., 2017b	2b	Induction of clinical remission at week 8, defined as FMS ≤ 2	7 mg^a^ (21)	1.6
				21 mg^a^ (40)	2.9
				70 mg^a^ (98)	13.5^∗^
				210 mg^b^ (79)	13.4^∗^
				Placebo (116)	4.4
AJM300 (p.o)	Yoshimura et al., 2015	2	Clinical response at week 8, defined as decrease of at least three points and 30% of FMS from baseline	960 mg three times daily (51)	62.7^∗^
				Placebo (51)	25.5
Etrolizumab (s.c.)	Vermeire et al., 2014	2	Induction of clinical remission at week 10, defined as FMS ≤ 2	100 mg^c^ (39)	21^∗^
				420/300 mg^d^ (39)	10^∗^
				Placebo (41)	0
PF-00547659 (s.c.)	Reinisch et al., 2015	2	Induction of clinical remission at week 12, defined as FMS ≤ 2	7.5 mg^c^ (NA)^e^	11.3^∗^
				22.5 mg^c^ (NA)	16.7^∗^
				75 mg^c^ (NA)	15.5^∗^
				225 mg^c^ (NA)	5.7
				Placebo (NA)	2.7
Tofacitinib (p.o)	Sandborn et al., 2016c	3	Induction of clinical remission at week 8, defined as FMS ≤ 2	10 mg twice daily (905)^f^	18^∗^
				Placebo (234)^f^	8.2
	Sandborn et al., 2017d	3	Maintenance of clinical remission at week 52, defined as FMS ≤ 2	5 mg twice daily (198)	34.3^∗^
				10 mg twice daily (197)	40.3^∗^
				Placebo (198)	11.1


*^∗^*p* < 0.05; FMS, Full Mayo Score; NA, not available; p.o, *per os* (oral); s.c., subcutaneous; i.v., intravenous; ^a^at week 0, 2, and 4, and then every 4 weeks; ^b^single dose; ^c^at week 0, 4, and 8; ^d^420 mg at week 0, and then 300 mg at week 2, 4, and 8; ^e^total number of patients: 357; ^f^OCTAVE 1 + OCTAVE 2.*

## Drugs that Interfere with Immune Cell Homing

After leaving the secondary lymphoid tissues, T lymphocytes reach, through the blood circulation, the tissues where the initial antigen stimulation arose. The first step of tissue homing is the “capture” of lymphocytes by vascular endothelial cells and their “rolling” on the endothelium itself. This process takes place through the interaction between some proteins expressed in the plasma membrane of endothelial cells, called selectins, and their ligands expressed on lymphocyte surface. Then, chemokines, produced by the endothelial cells, activate the lymphocytes. The process of “activation” includes the change of conformation of the integrins, which are receptor molecules expressed in the plasma membrane of lymphocytes, thereby able to bind to their ligands expressed in the plasma membrane of the endothelial cells. These interactions allow the lymphocytes to stop and adhere to the endothelial cells. Transendothelial migration follows the processes of “arrest” and “adhesion” by the combined actions of chemokines and integrins. The adhesion phase has been the most fruitful so far, as potential target for pharmacological interventions. The adhesion of lymphocytes to the endothelial cells occurs through the interaction of integrins with their specific ligands, which are proteins belonging to the immunoglobulin gene superfamily expressed on the surface of endothelial cells. Integrins are components of the “catalytic receptors” family, more commonly known as type III receptors or kinase-linked receptors (Alexander et al., 2015). They are heterodimeric macromolecules composed by non-covalently joined α and β subunits, each of which spans once the plasma membrane (1TM receptors). Integrins can also bind to proteins of the extracellular matrix, such as fibronectin, laminin, or collagen. They have short intracellular domains through which connect counter-receptors or extracellular matrix proteins to the actin cytoskeleton (Morse et al., 2014). The receptor stimulation induces the activation of some kinases, such as in T cells, focal adhesion kinase, the Src family tyrosine kinases Src and Lck, and the Syk family tyrosine kinase ζ-chain-associated protein kinase of 70 kDa (Verma and Kelleher, 2014; Mitroulis et al., 2015). Then, the integrins bind to cytoskeletal proteins, in particular paxillin, vinculin, and actinin (Mitroulis et al., 2015). The most important integrins expressed in lymphocytes are α_L_β_2_ (also known as Cluster of Differentiation [CD] 11a/CD18 [CD11a/CD18], or lymphocyte function-associated antigen 1), α_4_β_1_ (CD49d/CD29), and α_4_β_7_ (CD49d/β_7_). They specifically bind to some cell adhesion molecules, in particular: α_L_β_2_ to Intercellular Adhesion Molecule-1 and -2 (CD54 and CD102, respectively), α_4_β_1_ to VCAM-1 (or CD106), fibronectin and other extracellular matrix proteins, and α_4_β_7_ to MAdCAM-1 and VCAM-1. By binding to MAdCAM-1, the integrin α_4_β_7_ drives selectively the homing of lymphocytes to gastrointestinal tissues. Conversely, the integrin α_4_β_1_ is involved in lymphocyte homing to most organs and tissues. Two monoclonal antibodies are available on the market targeting lymphocyte integrins. **Natalizumab** is a recombinant humanized anti-α4 subunit monoclonal antibody belonging to the class of IgG4κ, for i.v. use, approved for the treatment of patients with highly active relapsing remitting MS who did not get any benefit from at least one disease modifying treatment, and CD, as second line therapy in the USA. By binding to the integrins α_4_β_1_ and α_4_β_7_ on lymphocyte plasma membrane, natalizumab inhibits lymphocyte homing to most organs and tissues. Although randomized placebo-controlled trials demonstrated the clinical efficacy of natalizumab in CD patients (Ley et al., 2016), several concerns have been raised about safety, related to the increased risk of developing PML. This condition is a severe brain inflammation resulting from reactivation of latent infection with the JC virus. PML has an incidence of approximately 2 in 1,000 MS patients treated with natalizumab for longer than 2 years. Due to this potentially fatal complication, the USA FDA withdrew temporally natalizumab from the market in 2005 and subsequently readmitted it under a controlled program for risk management in MS and CD patients. Of the same class, **vedolizumab**, a monoclonal humanized IgG_1_ antibody selectively directed against an epitope formed by the dimer α_4_β_7_, is approved for the treatment of both UC and CD. The safety profile of vedolizumab appears more favorable due to the gut selective mode of action, with no cases of PML reported so far.

A new anti-α_4_β_7_ antibody, **abrilumab** (AMG 181 or MEDI7183; **Table 1**), a human monoclonal IgG_2_, is in clinical development. Similar to vedolizumab, abrilumab binds with high affinity (*K*_D_ = 9 pM) to α_4_β_7_, but not α_4_β_1_; consequently, it specifically inhibits the binding of α_4_β_7_ to MAdCAM-1 (Pan et al., 2013a). In cynomolgus monkeys, exposure to abrilumab after i.v. or s.c. administrations is related to percentage binding to α_4_β_7_, increase in peripheral counts of gut-homing CD4^+^ T cells and immunogenicity (Pan et al., 2013a). Abrilumab shows dose-proportional C_max_s and AUCs following single s.c. administrations in the dose range 21–210 mg and 70–210 mg, respectively, in healthy men (Pan et al., 2014). Bioavailability of s.c. administered abrilumab and its t_1/2_ of the linear β-phase are approximately 82–99% and 31 days, respectively (Pan et al., 2014). Abrilumab pharmacokinetics is non-linear at plasma concentrations ≤1 μg⋅mL^-1^, consistent with target-mediated disposition (Pan et al., 2014). Abrilumab binding to α_4_β_7_ shows an EC_50_ of approximately 0.01 μg⋅mL^-1^. A single dose of 210 mg abrilumab produces α_4_β_7_ occupancy > 90% for longer than 127 days in CD4^+^ naive and central memory T cells; it also decreases total α_4_β_7_ levels on these cells by 40–60% at nadir (Pan et al., 2014). All these effects on T cells were reversible. Similar pharmacokinetic and pharmacodynamics features were found in two out three patients with UC (Pan et al., 2014). Abrilumab has proven efficacy for UC patients in a phase 2b multi-dose induction study. Overall, 354 patients were randomized to receive s.c. abrilumab 7, 21, or 70 mg on day 1, week 2 and 4, and then every 4 weeks, or 210 mg on day 1, or matching placebo. The primary endpoint was clinical remission (FMS ≤ 2 with no sub-score > 1; Silverberg et al., 2005) at week 8, that was met in a significantly greater percentage of patients in 70 mg and 210 mg groups (13.5 and 13.4%, respectively) compared to placebo (4.4%; **Table 2**). Regarding the subgroup of anti TNF-α experienced patients, treatment with abrilumab 210 mg was superior to placebo across all endpoints (clinical remission, clinical response, and MH at week 8; Sandborn et al., 2017b). In parallel, abrilumab was also tested in patients with moderately to severely active CD in a phase 2b multi-dose trial. In this study, abrilumab was not superior to placebo in inducing clinical remission at week 8 (primary endpoint; **Table 3**), but showed a significant gain over placebo in terms of clinical response and remission, above all for anti TNF-α experienced patients (Sandborn et al., 2017a).

**Table 3:** Summary of clinical trials for new drugs in clinical development for moderate-to-severe Crohn’s disease.

Drug (route of administration)	Study reference	Study phase	Primary endpoint	Drug dose (No. of patients)	% of patients who achieved the primary endpoint
Abrilumab (s.c.)	Sandborn et al., 2017a	2b	Induction of clinical remission at week 8, defined as CDAI < 150 points	21 mg^a^ (26)	23.1
				70 mg^a^ (84)	14.4
				210 mg^b^ (41)	21.9
				Placebo (98)	12.8
AJM300 (p.o)	Takazoe et al., 2009	2	Clinical response evaluated by means of the mean decrease of CDAI (±SD) from baseline at week 4 or later	40 mg three times daily (NA)^c^	19.9 ± 74.1
				120 mg three times daily (NA)^c^	25.5 ± 61.3
				240 mg three times daily (NA)^c^	21.6 ± 84.9
				Placebo (NA)^c^	5.2 ± 71
PF-00547659 (s.c.)	Sandborn et al., 2015	2	Clinical response at week 12, defined as reduction of CDAI ≥ 70 points	22.5 mg^d^ (67)	62
				75 mg^d^ (64)	65
				225 mg^d^ (68)	58
				Placebo (63)	59
Risankizumab (i.v.)	Feagan et al., 2016c	2	Induction of clinical remission at week 12, defined as CDAI < 150 points	200 mg^d^ (41)	24.4
				600 mg^d^ (41)	36.6^∗^
				Placebo (39)	15.4
Brazikumab (i.v.)	Sands et al., 2015	2a	Clinical response at week 8, defined as reduction of CDAI ≥ 100 points	700 mg^e^ (57)	49.2^∗^
				Placebo (55)	26.7
PF-04236921 (s.c.)	Danese et al., 2016	2	Clinical response at week 12, defined as reduction of CDAI ≥ 70 points	10 mg^e^ (65)	35.2
				50 mg^e^ (68)	47.4^∗^
				Placebo (69)	28.6
Filgotinib (p.o)	Vermeire et al., 2017a	2	Induction of clinical remission at week 10, defined as CDAI < 150 points	200 mg once daily (128)	48^∗^
				Placebo (44)	23
Mongersen (p.o)	Monteleone et al., 2015	2	Induction of clinical remission at day 15, defined as CDAI < 150 points	10 mg once daily (41)	22
				40 mg once daily (40)	65^∗^
				160 mg once daily (43)	55^∗^
				Placebo (42)	10^∗^


*^∗^*p* < 0.05; CDAI, Crohn’s disease activity index; SD, standard deviation; NA, not available; p.o, *per os* (oral); s.c., subcutaneous; i.v., intravenous; ^a^at week 0, 2, and 4, and then every 4 weeks; ^b^single dose; ^c^total number of patients: 71; ^d^at week 0, 4, and 8; ^e^at week 0 and 4.*

Another drug in clinical development that targets a component of the integrin family is **AJM300** (**Table 1**). Similar to natalizumab, it binds to α_4_ subunit, inhibiting the interactions of α_4_β_1_ with VCAM-1 and α_4_β_7_ with MAdCAM-1 and VCAM-1. However, differently from natalizumab, it is not a monoclonal antibody, but a small molecule, suitable for oral administration. AJM300 is a phenylalanine derivative having a quinazolinedione ring (Kataoka et al., 2012) developed by the Japanese company Ajinomoto Pharmaceuticals. The latter has not disclosed information on the chemical structure and human pharmacokinetics of the compound yet. AJM300 seems to be a prodrug that is converted in an active metabolite, HCA2969, whose pharmacodynamics was studied *in vitro* (Sugiura et al., 2013). HCA2969 binds with high affinity to human α_4_β_1_ and α_4_β_7_ (with *K*_D_ of 0.32 and 0.46 nM, respectively), thereby inhibiting the binding of α_4_β_1_ to VCAM-1 and α_4_β_7_ to MAdCAM-1 with IC_50_ of 5.8 and 1.4 nM, respectively (Sugiura et al., 2013). AJM300 (0.3–300 mg/kg), administered orally to mice, dose-dependently inhibits lymphocyte homing to Peyer’s patches and increases the counts of peripheral lymphocytes. The first effect has been shown to be related to the plasma concentrations of the active metabolite HCA2969 (Sugiura et al., 2013). Oral AJM300 also significantly reduces the histopathological severity of colitis in the murine model of the adoptive transfer of IL-10 deficient CD4^+^ T cells (Sugiura et al., 2013). AJM300 has shown some evidences of efficacy and safety in UC and CD patients in induction phase 2 studies. As far as UC is concerned, Yoshimura et al. (2015) randomized 102 patients with moderate-to-severe active disease despite treatment with mesalamine or steroids, but naive to immunosuppressants or anti TNF-α drugs, to receive AJM300 (960 mg) three times daily or matching placebo for 8 weeks. AJM300 showed a significantly higher clinical response at week 8 compared to placebo (62.7 and 25.5%, respectively, *p* = 0.0002; **Table 2**). At the same time-point, there were significant differences over placebo in terms of clinical remission and MH (Yoshimura et al., 2015). Regarding CD, 71 patients with active disease (defined as a CD activity index [CDAI] > 150 points plus abnormal serum level of C-reactive protein [CRP]) were randomized to receive AJM300 at three different dosages (40, 120, and 240 mg) three times daily or placebo. The primary endpoint was the clinical response evaluated by the mean decrease of CDAI score from baseline to week 4 or later. No differences were found in terms of clinical response between patients in any of the active arms and patients in the placebo arm (**Table 3**). However, patients with a baseline CDAI ≧200, treated in the highest two regimens groups, seemed to benefit more from treatment (Takazoe et al., 2009). Finally, **firategrast** (SB683699) and **TRK-170**, binding α_4_ and α_4_β_7_ integrins, respectively, are also being evaluated in CD in phase 2 trials, but no results have been published so far (NCT00101946 and NCT01345799).

In this same class, two other drugs, having different molecular mechanisms, are being developed: etrolizumab (rhuMAb Beta7 or PRO145223) and PF-00547659. **Etrolizumab** (**Table 1**) is a humanized IgG_1_ anti-β_7_ (CD103) subunit monoclonal antibody, specifically reacting with the integrins α_4_β_7_ and α_E_β_7_. The latter binds to E-cadherin, a cell adhesion molecule specifically expressed in epithelial cells, involved in lymphocyte homing to the gastrointestinal wall (Smids et al., 2016). Similar to vedolizumab, etrolizumab inhibits the gastrointestinal homing of α_4_β_7_^+^ lymphocytes by interfering with the interaction between α_4_β_7_ and MAdCAM-1, but also decreases the gastrointestinal retention of α_4_β_7_^+^ lymphocytes by hindering the interaction between α_4_β_7_ and E-cadherin. Etrolizumab increases the levels of circulating β_7_^+^ mucosal-homing lymphocytes, but does not affect the counts of circulating β_7_^-^ peripheral-homing lymphocytes in cynomolgus monkeys (Stefanich et al., 2011). Consistent with these findings, it inhibits lymphocyte homing to the colonic mucosa of severe combined immunodeficiency mice with CD45RB^high^ CD4^+^ T cell transfer-induced colitis. In contrast, differently from an anti-α_4_ antibody, etrolizumab does not reduce disease and histological severity scores in mice with experimental autoimmune encephalitis (Stefanich et al., 2011). A phase 1 clinical trial investigated the pharmacokinetics, pharmacodynamics, and safety of etrolizumab in patients with moderate-to-severe UC (Rutgeerts et al., 2013). This study included single ascending dose and multiple dose sub-studies. In the first sub-study, etrolizumab was administered at the doses of 0.3–10 mg/kg i.v. or 3.0 mg/kg s.c. In the multiple dose sub-study, the antibody was administered at the doses of 0.5–3.0 mg/kg s.c. or 4.0 mg/kg i.v. every 4 weeks for three cycles. Etrolizumab showed pharmacokinetic features typical of humanized monoclonal antibodies and first-order absorption and elimination kinetics, fitting well with a 2-compartmental model. C_max_s and AUCs were dose-proportional, except for doses ≤ 1.0 mg/kg, due to target-mediated clearance (Rutgeerts et al., 2013). Consequently, the mean t_1/2_ was shorter at lower doses (approximately 5–7 days at 0.3–1.0 mg/kg) than at higher doses (approximately 11–13 days at 3.0–10 mg/kg; Rutgeerts et al., 2013). The bioavailability for s.c. administration was approximately 53–67% (Rutgeerts et al., 2013; Wei et al., 2015), and the estimated *V*_d_ was 3.2 L (Wei et al., 2015). In the multiple dose sub-study, the three administrations of etrolizumab every 4 weeks resulted in moderate drug accumulation over time, with accumulation ratios of approximately 1.2-fold and 2.0-fold for i.v. and s.c. administrations, respectively (Rutgeerts et al., 2013). Only 2 out of 38 etrolizumab-treated patients showed the occurrence of plasmatic anti-etrolizumab antibodies. Etrolizumab occupied approximately all β_7_-containing integrins on mucosal-homing CD45RA- β_7_^high^ CD4^+^ T cells at all dosed tested (Rutgeerts et al., 2013). Occupancy duration was approximately 2–4 weeks and 8–12 weeks after last 0.5–1.5 mg/kg or 3.0 mg/kg s.c. dose, respectively, in the multiple dose sub-study (Rutgeerts et al., 2013). It was calculated that mean minimum serum concentrations of 0.5 and 1.7 μg⋅mL^-1^ are needed to maintain the full β_7_-containing integrins occupancy on mucosal-homing CD45RA- β_7_^high^ CD4^+^ T cells, and in peripheral blood and colonic tissues, respectively (Rutgeerts et al., 2013; Wei et al., 2015). Etrolizumab did not induce any dose limiting unwanted effect nor infusion or injection site reactions (Rutgeerts et al., 2013). Two serious AEs occurred in two patients receiving etrolizumab 1 mg/kg i.v. or 3 mg/kg s.c. in the single ascending dose sub-study, consisting in impaired wound healing following urgent colectomy. In two patients, JCV shedding into the serum was detected at low copy numbers per ml, with no evidence of PML symptoms. Patients treated with etrolizumab complained more often of headache and nausea (Rutgeerts et al., 2013). The efficacy and safety of etrolizumab in UC was evaluated in a phase 2 dose-finding, double blind, vs. placebo study, including 124 patients with moderately-to-severely active disease, who had previously failed conventional therapies. These patients were randomized (1:1:1) to receive either two different induction regimens of s.c. etrolizumab (100 mg at week 0, 4, and 8 or 420 mg at week 0, and then 300 mg at week 2, 4, and 8, respectively) or matching placebo. Clinical remission at week 10 (primary endpoint), defined as FMS ≤ 2 with no subscore > 1, was met in 21 and 10% of etrolizumab groups vs. 0% in placebo arm (*p* = 0.0040 and *p* = 0.048, respectively; Vermeire et al., 2014). Patients who completed through week 10 but did not reach clinical response or relapsed between week 10 and week 28, could enter the OLE, aimed to evaluate the long-term safety of etrolizumab. In this recently completed study, patients received 100 mg of etrolizumab every 4 weeks up to 240 weeks (NCT01461317); data are not available yet. The encouraging results of phase 2 study prompted a phase III trial, investigating the efficacy and safety of etrolizumab as induction and maintenance therapy in UC (HICKORY study, NCT02100696). To be eligible, patients had to be refractory or intolerant to anti TNF-α antibodies. In this study, two experimental cohorts were consecutively enrolled: the first (Cohort 1) receiving open-label 105 mg s.c. etrolizumab injections every 4 weeks for 14 weeks and the second one (Cohort 2) receiving double-blind 105 mg etrolizumab or matching placebo every 4 weeks up to week 66. A total of 130 patients were included in Cohort 1 and evaluated for symptomatic improvement, based on patients reported outcomes, including rectal bleeding and stool frequency. Most patients (80%) had a severely active endoscopic disease at baseline and almost all of them previously experienced ≥ 2 anti TNF-α. Both patients reported outcomes’ scores improved through week 14, with a mean (±SE) decrease in patients reported outcomes of 22% (±3%) at week 4 and 36% (±3%) at week 14 with a good correspondence with mean reductions in fecal calprotectin and CRP (Peyrin-Biroulet et al., 2017b). The underway double-blind induction part and maintenance phase should provide a better understanding of the drug’s efficacy and safety profile.

**PF-00547659** (**Table 1**) is a fully human IgG_2k_ anti-MAdCAM-1 monoclonal antibody that inhibits the interaction of this cell adhesion molecule with α_4_β_7_. The affinity of PF-00547659 for human MAdCAM-1 was initially estimated to be very high (*K*_D_ of 16.1 pM; Pullen et al., 2009). The real *in vivo* affinity of PF-00547659 for MAdCAM-1 was subsequently shown to be much lower than that measured under the *in vitro* conditions used by Pullen et al. (2009), and the *K*_D_ of this antibody was calculated to be approximately 528–596 pM (Wang et al., 2017). In immunohistochemical experiments, PF-00547659 specifically stained the endothelial cells of human gastrointestinal and associated lymphoid tissues. It also stained endothelial cells in liver, lung, urinary bladder, and uterus in one out of three tested human samples, but not those of cerebellum, cerebral cortex, choroid plexus, and spinal cord (Pullen et al., 2009). PF-00547659 (1 mg/kg i.v.) significantly increased by approximately 1.5-fold the peripheral counts of total lymphocytes, CD3^+^, CD4^+^, and CD8^+^ T cells, and CD20^+^ B cells, but not those of CD16^+^ NK cells in cynomolgus monkeys (Pullen et al., 2009). At this dose, PF-00547659 also significantly increased the peripheral counts of β_7_^+^ naive, central and effector memory T-cell by approximately 2-, 3- and 2.5-fold, respectively, but not those of β_7_^-^ central memory T cells, consistently with the mechanism of action of this antibody (Pullen et al., 2009). PF-00547659 pharmacokinetics was investigated in a phase 1 double-blind placebo-controlled trial including six single-dose cohorts (i.v. 0.03–10 mg/kg and s.c. 3.0 mg/kg) and five multiple-dose cohorts (i.v. 0.1–3.0 mg/kg and s.c. 0.3–1 mg/kg; Martin et al., 2009). The *V*_d_ of PF-00547659 was approximately 5 L, as expected for a monoclonal antibody. Similar to other antibodies targeting membrane-bound macromolecules, PF-00547659 showed two elimination pathways: the target mediated disposition, due to the binding of the antibody to MAdCAM-1 with subsequent internalization and elimination, and a linear pathway, with t_1/2_ of approximately 4 weeks (Martin et al., 2009). The first pathway was predominant at lower doses and saturated at doses ≥ 1 mg/kg. Following single doses of 3–10 mg/kg, sustained plasma concentrations were observed, and full MAdCAM-1 occupancy occurred for 10–12 weeks in most subjects (Martin et al., 2009). PF-00547659 has completed phase 2 trials in IBD, showing encouraging results. In the multi-dose RCT TURANDOT, 357 patients with moderate-to-severe UC were randomized to receive PF-00547659 at four different dosages (7.5, 22.5, 75, or 225 mg s.c.) at week 0, 4, and 8 or matching placebo. The primary endpoint was clinical remission at week 12, defined as FMS ≤ 2. The outcomes of 7.5, 22.5, and 75 mg arms were statistically significant in favor of PF-00547659 compared to the placebo arm (**Table 2**). Furthermore, patients treated with PF-00547659 22.5 mg or 75 mg were more likely to achieve MH (Reinisch et al., 2015). Regarding CD, in the OPERA study, neither 70-CDAI response rates (primary endpoint) nor clinical remission rates of patients treated with PF-00547659 at week 12 were statistically different from those of patients receiving placebo (**Table 3**). However, patients with serum CRP baseline levels higher than the median value seemed more benefit from treatment with PF-00547659 (Sandborn et al., 2015). Data on maintenance phases for both studies are not available so far (NCT01771809 and NCT01298492).

## Drugs that Inhibit the Activation of Th17 Cell Pathway

Interleukin-12 and IL-23 are heterodimeric proteins constituted by the association of a common subunit, IL-12B β-chain (p40 subunit), with either IL-12A α-chain (p35 subunit) or IL-23A α-chain (p19 subunit), respectively (Brocker et al., 2010; Vignali and Kuchroo, 2012). They are pro-inflammatory cytokines that induce Th1 and Th17 cell pathways, respectively. Moreover, the IL-23 pathway seems to be particularly involved in the pathogenesis of CD. Ustekinumab, a fully human monoclonal anti-p40 IgG_1κ_ antibody, is already available for the treatment of CD patients and under investigation for UC. Two molecules, both targeting the p19 subunit of IL-23, are in clinical development: risankizumab (BI 655066 or ABBV-066) and brazikumab (AMG 139 or MEDI2070). **Risankizumab** (**Table 1**) is a fully human IgG_1_ anti-p19 subunit monoclonal antibody. A first-in-human proof-of-concept study with risankizumab was carried out in patients with moderate-to-severe plaque psoriasis (Krueger et al., 2015). In this double blind, phase 1 RCT, 47 patients received either a single dose of risankizumab (i.v. at six different rising doses from 0.01 to 5 mg/kg, or s.c. 0.25 or 1 mg/Kg), or matching placebo. The AUCs were dose-proportional, indicating that the drug follows a linear first-order pharmacokinetics; the *V*_d_ was 10.8 L, and the t_1/2_ ranged from 20 to 28 days (Krueger et al., 2015). After s.c. administration, the bioavailability was approximately 59%, and maximal exposures were reached after 4–10 days (Krueger et al., 2015). Risankizumab was well tolerated over 24 weeks after the administration. AEs were observed in 65% of patients receiving risankizumab with respect to 88% of those receiving placebo. Nasopharyngitis, headache, and upper respiratory tract infections were the most frequent AEs in patients treated with risankizumab. Plasma anti-drug antibodies were detected only in two patients, with no apparent association with loss of response or hypersensitivity reactions (Krueger et al., 2015). With regard to IBD, first data coming from phase 2 study in CD patients were recently presented (Feagan et al., 2016a,b). This study consists of three different parts: (1) an i.v. induction phase aimed to evaluate clinical and endoscopic remission at week 12; (2) a 14-week, open-label i.v. re-induction/washout period; (3) a 26-week s.c. maintenance period. In total, 121 patients with clinically and endoscopic proven active CD, were initially randomized to receive either i.v. 200 or 600 mg risankizumab, or placebo, at weeks 0, 4, and 8. Risankizumab, at a dose of 600 mg, showed significantly higher clinical and endoscopic remission rates at week 12 compared to placebo (36.6% vs. 15.4%, *p* = 0.025, and 19.5% vs. 2.6%, *p* = 0.017, respectively; **Table 3**). After the first 12 weeks, patients entered the second part: in case of deep remission (clinical and endoscopic) underwent a 14-week wash-out, or otherwise an open-label i.v. risankizumab 600 mg re-induction (three infusions at week 14, 18, and 22). No patients with deep remission relapsed during the risankizumab washout phase to week 26. Clinical remission rates at week 26 were similar in re-induced patients, regardless of the original first part treatment designation. In terms of safety, risankizumab was well tolerated with similar AEs rates across the groups (Feagan et al., 2016c). In the same study, the effects of the drug on a transcriptome-wide profiling by RNA sequencing were investigated. At week 12, risankizumab significantly reduced the expression of genes associated with the IL-23 pathway, innate immunity, tissue turnover and transporters belonging to the solute carrier family in the inflamed colon, but not in the inflamed ileum, of patients with clinical benefit (Visvanathan et al., 2016). **Brazikumab** (**Table 1**) is a human IgG_2_ monoclonal antibody that binds with high affinity the p19 subunit, with a *K*_D_ of 0.138 nM for human IL-23 (Köck et al., 2015). Brazikumab inhibits the binding of both recombinant and native IL-23 to its receptors stably expressed in COS cells with IC_50_ of 188 and 284 pM, respectively (Köck et al., 2015). Brazikumab inhibits the *in vitro* production of IFN-γ in NK cells stimulated by human native or recombinant IL-23 (in association with IL-18) with IC_50_ of 238 and 93 pM, respectively. Conversely, the drug did not affect the production of IFN-γ induced in NK cells by IL-12 (Köck et al., 2015). Brazikumab also reduced the IL-23 stimulated production of IFN-γ in whole human blood with an IC_50_ of 28 pM (Köck et al., 2015). Pharmacokinetics, pharmacodynamics, and safety of brazikumab were studied in two phase 1 double blind RCT (Pan et al., 2013b). In these studies, healthy volunteers received single or multiple ascending doses of brazikumab (s.c. 7–210 mg or i.v. 210–420 mg in the single dose sub-study, and i.v. 70–700 mg or s.c. 210 mg once a month for three times in the multiple dose sub-study), or matching placebo. S.c. brazikumab showed a linear pharmacokinetics in the dose range 21–210 mg, with a t_1/2_ of 22–37 days; bioavailability was 64–86% (Pan et al., 2013b). After multiple administrations, brazikumab plasma concentrations progressively increase, reaching C_max_s after the third dose that were 1.5 to 2.1 times higher than those observed after the first dose (Pan et al., 2013b). Brazikumab did not induce any serious AE or the formation of anti-drug antibodies in either study (Pan et al., 2013b). Brazikumab is in phase 2b of clinical development for CD (NCT02574637). In the induction phase 2a study, brazikumab displayed clear efficacy for anti TNF-α refractory moderate-to-severe CD. Overall, 121 patients, stratified by number of prior anti-TNF agents, were randomized to i.v. brazikumab 700 mg or matching placebo at weeks 0 and 4. The primary outcome was week 8-clinical effect, defined as either clinical response (≥100-point drop from baseline CDAI) or clinical remission (CDAI < 150). Brazikumab showed greater clinical benefit than placebo in terms of both clinical effect (49.2% vs, 26.7%, respectively, *p* = 0.010) and clinical response (45.8% vs. 25.0%, respectively (*p* = 0.017; **Table 3**). No increased rate of AEs with active treatment was observed along the study as compared to placebo (Sands et al., 2015).

## Drugs that Inhibit the Activation of IL-6 Pathway

Interleukin-6 is another pro-inflammatory cytokine involved in IBD pathophysiology. **PF-04236921** (**Table 1**) is a fully human IgG_2_ anti-IL-6 monoclonal antibody, for s.c. use, in clinical development for anti TNF-α refractory moderate-to-severe CD patients. Pharmacokinetics, pharmacodynamics, and safety of PF-04236921 were studied in two phase 1 double blind RCTs (Fogel et al., 2014). In the first trial, healthy volunteers received i.v. PF-04236921 at one out of seven doses in the range 7–700 mg, or placebo. In the second trial, healthy volunteers received PF-04236921 200 mg s.c. or placebo. Plasma C_max_s and AUCs increased in approximately dose-dependent manner following i.v. administration of ascending doses. A bicompartmental pharmacokinetic model better described the AUCs, with t_1/2_ of the β phase of approximately 51 days and central and peripheral compartment volumes of approximately 3.2 L and 3.7 L, respectively. The s.c. bioavailability was >90%. The antibody decreased CRP serum levels, with an EC_50_ of 422 ng⋅mL^-1^, and transiently blood neutrophil counts, and increased the serum concentrations of IL-6. PF-04236921 did not induce the formation of anti-drug antibodies. Three serious AEs occurred in two subjects in the i.v. ascending dose study. The preliminary results of the phase 2 trial, in which 247 CD patients were randomized to receive PF-04236921 (10, 50, or 200 mg) or placebo at week 0 and 4, showed a significant clinical response in the 50-mg group vs. placebo at both week 8 and 12 (49.3% vs. 30.6%, and 47.4% vs. 28.6%, respectively, *p* < 0.05; **Table 3**). The clinical remission rate at week 12 was also significantly greater in the 50-mg group compared to placebo (27.4% vs. 10.9%, respectively, *p* < 0.05). The 200-mg arm was interrupted early due to safety concerns. In the remaining groups, PF-04236921 globally appeared to be quite well tolerated (Danese et al., 2016).

## Drugs that Inhibits the Signal Transduction Pathways Activated by Cytokines

The JAKs are four intracellular tyrosine kinases (JAK1, JAK2, JAK3 and TYK2) that are part of the signaling pathways of some catalytic receptors (Banerjee et al., 2017). They phosphorylate the transcription factors belonging to the STAT family, which includes seven members (STAT1-4, 5a, 5b, and 6; Banerjee et al., 2017). The JAK-STAT signaling pathway is activated by receptors for class I and II helical cytokines (Brocker et al., 2010), and some hormones, such as erythropoietin, thrombopoietin, growth hormone, prolactin, and leptin. The first group of molecules includes many ILs (except for IL-1-like and IL-17-like ILs, IL-8, IL-14, IL-16, IL-32, and IL-34), IFNs, GM-CSF, and other cytokines specified below. Most cytokine receptors are dimeric, and a JAK molecule is bound to the intracellular domain of each subunit. Following the binding of cytokines, the receptors change their conformation and the intracellular domains of the subunits, with the associated JAKs, move apart and separate, with the consequent activation of JAKs (Banerjee et al., 2017). Activated JAKs phosphorylate themselves and the intracellular domains of the receptor subunits, which, in phosphorylated state, serve as docking sites for STATs (Banerjee et al., 2017). The latter, bound to the receptors, are in turn phosphorylated by JAKs, change the molecular conformation, and dimerize. Then, STAT dimers dissociate from receptors and translocate to the nucleus, where they carry out their function of transcription factors (Banerjee et al., 2017). Receptors for different cytokine classes are associated with different JAKs and, in turn, different JAKs phosphorylate different sets of STATs (Roskoski, 2016; Zundler and Neurath, 2016; Banerjee et al., 2017).

Based on genomic architecture and protein structures, class I cytokines are classified as short- or long-chain cytokines (Brocker et al., 2010). Short-chain class I cytokines are divided into γ-chain utilizing and IL-4-like cytokines; IL-4 belongs to both groups (Brocker et al., 2010). IL-2, IL-4, IL-7, IL-9, IL-15, IL-21 and thymic stromal lymphopoietin bind to heterodimeric or heterotrimeric receptors containing the γ-chain, which associates only with JAK3 (Roskoski, 2016; Zundler and Neurath, 2016; Banerjee et al., 2017). The other receptor subunits, which are each specific for each different IL of this group, associate with JAK1. IL-4-like cytokines include IL-4 and IL-13, the receptors of which share the IL-4Rα subunit, and IL-3, IL-5, and GM-CSF, the receptors of which share the β-chain. Both the β-chain and the other selective subunits composing the heterodimeric receptors for IL-3, IL-5, and GM-CSF associate with JAK2. The homodimeric receptors for the hormones are thought to associate only with JAK2 as well (Roskoski, 2016; Zundler and Neurath, 2016; Banerjee et al., 2017). The long-chain class I cytokines includes IL-6-like and IL-12-like cytokines. The IL-6-like cytokines are monomeric and include IL-6, IL-11, IL-31, granulocyte colony-stimulating factor, ciliary neurotrophic factor, cardiotrophin 1, cardiotrophin-like cytokine factor 1, leukemia inhibitory factor, oncostatin M and thymic stromal lymphopoietin. These cytokines, with the exception of IL-31, bind to heterodimeric or heterotrimeric receptors sharing the IL6ST (gp130) subunit, which are associated with JAK1, JAK2, or TYK2 (Roskoski, 2016; Zundler and Neurath, 2016; Banerjee et al., 2017). The IL-12-like cytokines are heterodimeric proteins that include IL-12, IL-23, IL-27, and IL-35. IL-12 and IL-23 are the main members of this group; their receptors are heterodimers composed by the IL-12Rβ1 subunit with either the IL-12Rβ2 or the IL-23R subunit, respectively (Brocker et al., 2010; Vignali and Kuchroo, 2012). IL-12Rβ1 subunit associates with TYK2, whereas IL-12Rβ2 and IL-23R subunits associates with JAK2 (Vignali and Kuchroo, 2012; Banerjee et al., 2017). Class II cytokines include the group of IL-10-like cytokines, and the group of IFNs and IL-28-like cytokines. The IL-10-like cytokines, which include IL-10, IL-19, IL-20, IL-22, IL-24, and IL-26, bind to receptor subunits associated with JAK1, JAK2, or TYK2 (Zundler and Neurath, 2016; Banerjee et al., 2017). The receptor subunits of type I IFNs associate with JAK1 and TYK2, whereas those of type II IFNs associate with JAK1 and JAK2 (Banerjee et al., 2017). The receptors for IL-28-like cytokines (IL-28A, IL-28B, and IL-29) associate with JAK1 and TYK2 (Zundler and Neurath, 2016; Banerjee et al., 2017).

Downstream to JAKs, different cytokines activate the several STATs. The picture is not so simple to depict, since many receptors are associated with more than one STAT and the JAK/STAT signaling activated by different cytokines can overlap. However, it is possible to highlight, simplifying, a few significant points. The most important role of STAT1 is to mediate the responses to type II IFN, whereas impaired responses to type I IFNs are detected in both STAT1 and STAT2 knockout mice (O’Shea et al., 2015). STAT3 is the main signal transducer of IL-6-like and IL-10-like cytokines (O’Shea et al., 2015; Banerjee et al., 2017). STAT4 and STAT5 are the main STATs activated by IL-12-like and IL-2-like cytokines, respectively, whereas STAT6 is the only mediator of IL-4- and IL-13-induced signaling (O’Shea et al., 2015; Banerjee et al., 2017). Mutations of STAT4 and STAT6 inhibit Th1 and Th2 differentiation, respectively (O’Shea et al., 2015). All STATs seem to be involved in the signaling of IL-27 and IL-28-like cytokines (Banerjee et al., 2017). In addition, STAT1 also participates to the signal transduction of IL-6-like cytokines and IL-35, whereas STAT3 is also involved in the signaling pathways activated by IL-2-like cytokines and IL-23 (O’Shea et al., 2015; Banerjee et al., 2017). STAT4 is also important for signaling by type I IFNs, whereas STAT1 and STAT5 also contribute to the signaling pathways activated by IL-10 and IL-22 (O’Shea et al., 2015; Banerjee et al., 2017).

The JAK-STAT pathway is involved in the pathogenesis of several immune-mediated diseases, including IBD, RA and psoriasis, and seems to have a critical role in some hematologic proliferative disorders (O’Shea et al., 2013). Therefore, the inhibition of JAKs has been proposed as potential therapeutic target for these disorders. Two JAK inhibitors, ruxolitinib, and tofacitinib, are already available for clinical use. Ruxolitinib is approved for the treatment of patients with polycythemia vera who have had an inadequate response to or are intolerant of hydroxyurea, or with intermediate or high-risk myelofibrosis. **Tofacitinib** (CP-690,550; **Table 1**) is currently on the market for the care of patients with moderate-to-severe RA, as a second-line after failure of one or more disease-modifying anti-rheumatic drugs (Singh et al., 2016). The encouraging results of phase 3 OCTAVE program put potentially tofacitinib for the treatment of UC (Sandborn et al., 2016c). Conversely, for CD, in phase 2 study, tofacitinib failed the primary endpoint, not showing any significant difference compared to placebo (D’Haens et al., 2016; Panés et al., 2016). Tofacitinib is an orally administered small molecule that selectively inhibits JAKs. It inhibits the activity of human recombinant kinase domains of JAK1, JAK2, JAK3, and TYK2 with IC_50_ of 3.2, 4.1, 1.6, and 34 nM, respectively (Meyer et al., 2010), and binds to human JAK2 and JAK3 with *K*_D_ of 5 and 2.2 nM, respectively (Karaman et al., 2008). In human CD3^+^ T lymphocytes, tofacitinib inhibits the phosphorylation of STAT3 and STAT5 by some γ-chain utilizing cytokines (IL-2, IL-7, and IL-21), STAT6 by IL-4 (JAK1/JAK3-dependent), STAT1 and STAT3 by IL-6 (JAK1/JAK2/TYK2-dependent), and STAT1 by IFN-α (JAK1/TYK2-dependent), with IC_50_s of 25–38 nM, 50 nM, 54–367 nM, and 44 nM, respectively (Meyer et al., 2010). In human monocytes, the drug inhibits the phosphorylation of STAT1 by IFN-α and IFN-γ (JAK1/TYK2- and JAK1/JAK2-dependent, respectively), STAT3 by IL-6 (JAK1/JAK2/TYK2-dependent), and STAT5 by GM-CSF (JAK2-dependent), with IC_50_s of 158–178 nM, 406 nM, and 1,377 nM, respectively (Meyer et al., 2010). Tofacitinib (0.1–1 μM) inhibits the differentiation of mouse naive Th cells in Th1 and Th2 cells, and interferes with Th17 cell differentiation as well (Ghoreschi et al., 2011). Tofacitinib (20–500 nM) also concentration-dependently inhibits the increase in gene expression and secretion of IL-4, IFN-γ, IL-17A, and IL-22, but not IL-2, in human CD4^+^ T cells stimulated with a CD3 monoclonal antibody (Migita et al., 2011). Thus, tofacitinib reduces the activity of multiple JAKs and inhibits the secretion and actions of a range of cytokines, decreasing both innate and adaptive immunity (Hirahara et al., 2016). Safety, tolerability, and pharmacokinetics of tofacitinib in humans were evaluated in a double blind, parallel group, phase 1 RCT (Krishnaswami et al., 2015). Healthy volunteers were randomized to receive single doses of tofacitinib (0.1–100 mg) or placebo. Tofacitinib is rapidly absorbed after oral administration, reaching peak plasma concentrations after approximately 0.5–1 h (Krishnaswami et al., 2015); its oral bioavailability is approximately 74% (Gupta et al., 2011). Systemic exposures to the drug, evaluated by C_max_s and AUCs, are dose-dependent. Tofacitinib is eliminated in the urine as parent drug for approximately 30% of the absorbed dose (Dowty et al., 2014). The remaining part of the drug is eliminated by hepatic metabolism through oxidation and *N*-demethylation, mainly catalyzed by the 3A4 isoform of the cytochrome P450 oxidative system, followed by glucuronidation (Dowty et al., 2014). The mean terminal t_1/2_ of tofacitinib is in the range 2.3–3.1 h after administrations of 3–100 mg single doses (Krishnaswami et al., 2015). Drug exposure increases by 37–43% and 123% in patients with mild-to-moderate and severe chronic kidney disease, respectively (Krishnaswami et al., 2014), and 65% in patients with moderate cirrhosis (Lawendy et al., 2014). These exposure increases were the result of the increases in t_1/2_ from 2.4 h in healthy volunteers to 2.8–2.9 h and 3.8 h in patients with mild-moderate and severe chronic kidney disease, respectively (Krishnaswami et al., 2014), and from 4.1 h in healthy volunteers to 5.4 h in patients with moderate cirrhosis (Lawendy et al., 2014). In the phase 1 trial with single doses of tofacitinib, the investigators did not observe any serious AE in any treatment group; nausea was the only treatment emergent AE more frequent in tofacitinib-treated subjects than in those receiving placebo (Krishnaswami et al., 2015). The analysis of white blood cell count with differential and lymphocyte subset (CD3^+^, CD4^+^, CD8^+^, CD16^+^, and CD19^+^ cells) did not show any significant difference in volunteers receiving single doses of tofacitinib with respect to those receiving placebo (Krishnaswami et al., 2015). In contrast, tofacitinib induced dose-dependent increases in B cell counts and reductions of NK cells in patients with moderate-to-severe chronic plaque psoriasis treated for 12 weeks (Valenzuela et al., 2015). In particular, the median changes of B cell and NK cell counts from baseline in patients receiving tofacitinib 2, 5, or 15 mg twice daily were +18%, +35.3%, and +42.9%, and -20%, -22.8%, and -34.3%, respectively (Valenzuela et al., 2015). The ability of tofacitinib to inhibit the actions of several cytokines and to reduce NK cell counts, affecting both innate and adaptive immune responses, has raised concerns about the increased risk of infection and cancer. In fact, the most frequently reported AEs of tofacitinib in RA patients are infections, particularly nasopharyngitis (12.7%) and upper respiratory tract infection (10.5%), also potentially related to the reductions of lymphocyte and neutrophil counts associated with the long-term treatment with the drug (Wollenhaupt et al., 2014). Patients treated with tofacitinib are at increased risk for developing serious infections, including herpes zoster, invasive fungal infections, tuberculosis, and other opportunistic infections (Wollenhaupt et al., 2014). Anemia and elevations of liver enzymes, serum creatinine and lipid levels can also occur in patients treated with tofacitinib (Wollenhaupt et al., 2014). Anemia seems to be related to the inhibition of JAK2, which transduces the signal of erythropoietin. It is still uncertain whether the increase in serum low-density lipoprotein cholesterol levels is associated with an increased rate of major cardiovascular AEs. Available data seem to exclude this risk, but further long-term studies are awaited (Charles-Schoeman et al., 2016). The long-term (up to 8.5 years) safety profile of tofacitinib is available from an integrated analysis of global clinical trial data in RA (Cohen et al., 2017). Data from a total 19,406 patient-years’ exposure and a median exposure of 3.4 patient-years receiving tofacitinib 1–30 mg twice daily or 20 mg once daily, as monotherapy or with background disease modifying anti-rheumatic drugs, were analyzed; most patients received the drug at the dosage of 5 or 10 mg twice daily. This pooled analysis confirmed infections as the most common AE, mainly nasopharyngitis, upper respiratory tract infections and urinary tract infections. Incidence rates, expressed as patients with event/100 patient-years, for serious AEs, serious infections, herpes zoster, opportunistic infections (excluding tuberculosis), tuberculosis, malignancies (excluding non-melanoma skin cancer), and non-melanoma skin cancer were 9.4, 2.7, 3.9, 0.3, 0.2, 0.9, and 0.6, respectively (Cohen et al., 2017). The most frequent serious infections and malignancies were pneumonia, herpes zoster, urinary tract infections, and cellulitis, and lung cancer, breast cancer, and lymphoproliferative disorders, including lymphoma, respectively. The incidence rate for lymphoproliferative disorders seems to be 2.6 times higher than that observed in the general population (Cohen et al., 2017). The OCTAVE program consisted of two identical RCTs (OCTAVE 1 and 2), each enrolling more than 500 patients with moderate-to-severe UC, who previously failed at least one among steroids, thiopurines or anti TNF-α (these last were experienced in more than 50% of them). In both studies, patients were randomized (4:1) to receive 10 mg tofacitinib twice daily or matching placebo along 8 weeks. Primary endpoint was week 8 clinical remission, which was achieved in significantly greater percentage of tofacitinib treated patients, with a therapeutic gain over placebo up to 13% (**Table 2**). At the same time-point, tofacitinib was significantly superior to placebo for inducing MH and clinical response (secondary endpoints). Tofacitinib showed a good safety profile, with AE rates comparable to placebo. However, elevation of serum cholesterol and creatine kinase levels were recorded (Sandborn et al., 2016c). At the end of 8 weeks, patients at least in clinical response could enter the OCTAVE Sustain study and re-randomized to receive maintenance therapy with tofacitinib 5 or 10 mg twice daily or matching placebo through 52 weeks. Clinical remission rate at week 52 (primary endpoint) was significantly greater in both active arms compared to placebo (34.3, 40.3, and 11.1%, respectively, *p* < 0.001 for both comparisons; **Table 2**). Tofacitinib showed a significantly greater effect vs. placebo also for key secondary endpoints, including MH at week 52 and sustained-free remission. AE rates were similar across the groups, even though significant increases in infection and herpes zoster rates were recorded in tofacitinib groups (Sandborn et al., 2017d).

**Filgotinib** (GLPG0634 or GS-6034; **Table 1**) is an oral JAK inhibitor with preferential inhibition of the signaling involving JAK1. It concentration-dependently inhibits human recombinant JAK1, JAK2, JAK3, and TYK2 with IC_50_ values of 10, 28, 810, and 116 nM, respectively, and binds to JAK1, JAK2, and JAK3 with *K*_D_ values that quite well correlate with IC_50_ values (11, 32, and 300 nM, respectively; Van Rompaey et al., 2013). In different *in vitro* cellular set-ups, the compound preferentially inhibits STAT phosphorylation mediated by JAK1 with respect to that mediated by JAK2 (Van Rompaey et al., 2013). In fact, filgotinib more potently inhibits the JAK1/JAK3-dependent phosphorylation of STAT6 and STAT5 induced by IL-4 or IL-2, respectively (with pIC_50_ of 6.75 and 6.46), than the JAK1/JAK2-dependent STAT1 phosphorylation and the JAK2-dependent STAT5 phosphorylation induced by oncostatin M or IL-3, respectively (with pIC_50_ of 6.01 and 5.34; Van Rompaey et al., 2013). The inhibition of STAT5 phosphorylation induced by erythropoietin or prolactin through the activation of JAK2 requires filgotinib concentrations ≥10 μM (Van Rompaey et al., 2013). In a human whole blood assay, filgotinib inhibited the phosphorylation of STAT1 induced by IL-6 in lymphocytes, which has been shown to be JAK1-dependent (Ghoreschi et al., 2011), with an IC_50_ (629 nM) 28 times lower than that (17.5 μM) with which it inhibited the JAK2-dependent phosphorylation of STAT5 in myeloid cells stimulated with GM-CSF (Van Rompaey et al., 2013). In the same assay, tofacitinib resulted 10 times more selective for JAK1 with respect to JAK2 (Van Rompaey et al., 2013). In addition, filgotinib concentration-dependently inhibits the differentiation of naive human CD4^+^ T cells into Th1, Th2, and Th17 cells following stimulation with anti-CD3/anti-CD28 antibodies (Van Rompaey et al., 2013). *In vivo*, filgotinib is hydrolyzed by hepatic carboxylesterases to an active metabolite, which has similar selectivity for JAK1 as the precursor molecule. This active metabolite produces a significant part of the overall pharmacological activity of the orally administered drug, even though it is approximately 17 times less potent than the parent compound in inhibiting JAK1 in the human whole blood assay (IC_50_ of 11.9 μM; Namour et al., 2015). Pharmacokinetics, pharmacodynamics, safety, and tolerability of filgotinib and its metabolite were investigated in two double blind, phase 1 RCTs in healthy male volunteers. In these studies, the volunteers received single doses, in the range 10–200 mg, or repeated doses (25–100 mg twice daily, or 200–450 mg once daily for 10 days) of filgotinib, or placebo (Namour et al., 2015). Filgotinib is rapidly absorbed after oral administration, with mean t_max_ values of 0.5–3 h. The increase in C_max_ and AUC of filgotinib were dose-dependent, and the mean t_1/2_ was 4.9–5.7 h over the anticipated therapeutic dose range of 50–200 mg (Namour et al., 2015). Within this dose range, the plasma concentrations of the active metabolite peaked 3–5 h after the drug administration and slowly decreased with a t_1/2_ of 18.1–22.5 h (Namour et al., 2015). Consequently, the exposures to the active metabolite were 16–20 times higher than those observed to filgotinib, and this higher exposure compensated for the lower potency of the metabolite (Namour et al., 2015). In these phase 1 studies, the authors also investigated STAT1 phosphorylation induced by IL-6 in the whole blood assay carried out after the administration of single doses or repeated administrations of filgotinib for 10 days, i.e., at the steady state. A sigmoid E_max_ model described adequately the relationship between plasma concentrations of filgotinib and its active metabolite and the inhibition of STAT1 phosphorylation, with IC_50_ of 293 ng⋅mL^-1^ for filgotinib and 1,686 ng⋅mL^-1^ for the metabolite. These values translate in a 5.7-time higher potency of filgotinib with respect to the metabolite, which is lower than the approximately 17 times higher potency measured in the human whole blood assay (Namour et al., 2015). The recently published, phase 2 FITZROY study examined the efficacy and safety of filgotinib for patients with moderate-to-severe disease CD (with proven mucosal lesions). Overall, 174 patients (stratified according to baseline CRP, steroids use and history of anti TNF-α exposure) were initially randomized (3:1) to 200 mg filgotinib once daily or placebo for 10 weeks (part 1). The proportion of patients in clinical remission at week 10 (primary endpoint) was significantly higher in filgotinib group compared to placebo (47% vs. 23%, respectively, *p* = 0.0077, **Table 3**; Vermeire et al., 2017a). At the end of the study part 1, according to week-10 clinical response, patients were assigned to receive maintenance treatment with either filgotinib 200, 100 mg once daily or placebo for further 10 weeks. After this period, among 200 mg week-10 responders, a good proportion of patients were still in clinical response (50–71%) or remission (67–79%; Vermeire et al., 2017b). With regard to safety, filgotinib showed a good profile, although rates of serious treatment-emergent AEs and infections rates were higher with filgotinib compared to placebo. Unlike tofacitinib, treatment with filgotinib, due to its JAK1 selectivity, is not associated with significant elevations of serum lipids or anemia, as confirmed also in rheumatic studies (Westhovens et al., 2016). **Upadacitinib** (ABT-494) is another JAK1 inhibitor that is being evaluated in phase 2 for both CD and UC (NCT02365649 and NCT02819635, respectively). **Peficitinib** is an orally administered, non-selective JAK inhibitor (albeit showing a moderate JAK3 selectivity) in clinical development for UC. However, in the multi-dose phase 2b study including refractory patients, peficitinib did not show significant effect in terms of clinical response compared to placebo at week 8 (Sands et al., 2017).

## Drugs that Amplify the Signaling of Transforming Growth Factor (TGF)-β

An imbalance between pro-inflammatory and counter-regulatory mechanisms seems to be responsible for the protraction and amplification of tissue-damaging immune response in IBD. TGF-β1, TGF-β2, and TGF-β3 are regulatory cytokines with important physiological and pathophysiological roles in several biological processes, including embryogenesis and morphogenesis, immune response, wound healing, inflammation, and carcinogenesis (Santibañez et al., 2011). They are potent anti-inflammatory cytokines, produced by several immune and not-immune cells, including macrophages, T cells, and epithelial and stromal cells of gut mucosa (Kmieć et al., 2017). TGF-βs inhibit immune-inflammatory signals and promote tissues repair by various mechanisms, such as the stimulation of T regulatory cell induction and epithelial cell proliferation, and the inhibition of Th1 and Th2 differentiation (Oh and Li, 2013; Kmieć et al., 2017). A major role in the regulation of immune response and inflammation is played by TGF-β1, which is the main isoform expressed in the immune cells. The receptors for TGF-β belong to the receptor serine/threonine kinase family of the catalytic receptor superfamily (Alexander et al., 2015). TGF-βs, which are dimeric molecules, stabilize the receptors in a heterotetrameric quaternary structure composed by two heterodimers of TGF-β receptor (TGFBR) 1 and TGFBR2 subunits (Heldin and Moustakas, 2016). TGFBR1 and TGFBR2 are found in the plasma membrane as monomers, homodimers, and heterodimers. TGF-β1 and TGF-β3 have higher affinity for TGFBR2 and bind first to this subunit; then, the complex recruits TGFBR1 (Heldin and Moustakas, 2016). In the tetrameric receptor assembly with the bound cytokine, the heterodimeric receptor units can transduce the signal independently, and TGFBR2 phosphorylates the co-assembled TGFBR1, activating it (Heldin and Moustakas, 2016). In turn, the phosphorylated TGFBR1 selectively binds and phosphorylates two cytosolic protein, Smad2 and Smad3, which are members of the receptor-associated Smad subfamily. Phosphorylated Smad2 and Smad3 lose affinity for TGFBR1, dissociate from it and bind the co-operating Smad, Smad4. Then, Smad complexes translocate to the nucleus, where regulate the transcription of selected genes (Santibañez et al., 2011). Among these genes, there are the two inhibitory Smads, Smad6 and Smad7. The latter is involved in the inhibitory regulation of TGF-β signaling (Santibañez et al., 2011). It recruits the Smad-specific E3 ubiquitin protein ligases Smad ubiquitination regulatory factors to TGFBR1, which induce receptor polyubiquitylation and endocytosis toward the degradative pathway, thus decreasing TGFBR density and TGF-β-mediated responses (Massagué, 2012). Abnormal reduction of the activity of TGF-β1 has been reported in the inflamed intestine of IBD patients, and has been related to the over-expression of Smad7 (Monteleone et al., 2001). **Mongersen** (GED0301; **Table 1**) is a synthetic phosphorothioate antisense 21-base single-strand DNA oligonucleotide that hybridizes with the sequence 107–128 of Smad7 mRNA, inducing its degradation (Monteleone et al., 2001). It is administered orally, with a pH-dependent terminal ileum and right colon delivery, due to a methacrylic acid–ethyl acrylate copolymer coat (Monteleone et al., 2015). The coating should allow getting topical drug effects in the distal gut, limiting or avoiding the systemic exposure to the oligonucleotide. To verify this, the absorption of mongersen was evaluated in a study on 15 CD patients receiving the oligonucleotide at the dose of 40, 80, or 160 mg once daily for 7 days. The drug plasma levels were measured at different times on days 1 and 7, and resulted below the assay detection limit (7.5 ng⋅mL^-1^) in all samples but one at day 7 (Monteleone et al., 2012). Preliminary efficacy and safety of mongersen for treating CD have been evaluated in a short-term phase 2 study, enrolling 166 patients with moderate-to-severe active disease (defined as CDAI score 220–400) and documented endoscopic or radiological involvement of terminal ileum and/or right colon within the last year. Patients were randomized to receive one of three different dosages of mongersen (10, 40, and 160 mg once daily), or placebo for 15 days and then followed-up for further 2 weeks. The primary endpoint was clinical remission (CDAI < 150 points) rate at day 15 maintained up to day 28, that was met in greater percentages of patients treated with higher doses compared to placebo (55%, 65% vs. 10%, respectively, *p* < 0.001 for both comparison). As far as safety is concerned, the AE rate was similar across groups, with a prevalence of mild events (Monteleone et al., 2015). A phase 3 trial is currently underway (NCT02596893), including also endoscopic outcomes, to investigate the long-term efficacy and safety of mongersen for treating CD.

## Discussion

**FIGURE 1:**
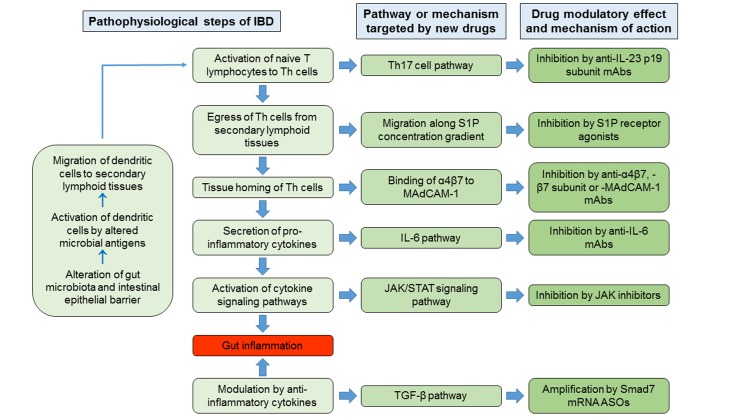
Schematic summary of the hypothesized primary pathophysiological steps of IBD, with corresponding pathways or mechanisms targeted by new drugs, and modulatory effects and mechanisms of action of new drugs. Th, T helper; S1P, sphingosine 1-phosphate; MAdCAM-1, Mucosal Vascular Addressin Cell Adhesion Molecule 1; IL, interleukin; JAK, Janus kinase; STAT, Signal Transducer and Activator of Transcription; TGF-β, transforming growth factor-β; mAbs, monoclonal antibodies; ASOs, antisense oligonucleotides.

Inflammatory bowel disease is chronic, progressive, and disabling diseases causing heavy morbidity and deterioration in quality of life for patients. The more and better comprehension of IBD natural history has induced a dramatic evolution of treatment paradigms, shifting from symptomatic control to more demanding outcomes, such as MH and deep remission (Sandborn et al., 2014). However, the management of patients with IBD remains a great challenge for health professionals, especially because currently available drugs show limited efficacy and have not been identified predictors of drug response. In addition, long-term use of immunomodulatory drugs has raised several safety concerns for the potential risk of infections and malignancies (Sandborn, 2016). Thus, there is a growing interest and need in exploring new potential drugs target. Recently, two new biological drugs, vedolizumab and ustekinumab, have been added to the therapeutic armamentarium for IBD. Vedolizumab is a monoclonal antibody, member of anti-integrin class, targeted against α4β7 integrin expressed on lymphocyte surface and interfering with lymphocyte homing to the intestinal mucosa, approved for treatment of both CD and UC (Feagan et al., 2013; Sandborn et al., 2013). Ustekinumab is a monoclonal antibody belonging to the big family of drugs inhibiting the activity of pro-inflammatory cytokines, already approved for treatment of CD and on clinical development (phase 3; NCT02407236) for UC (Feagan et al., 2016c). In particular, ustekinumab binds the subunit p40 shared by both IL-12 and IL-23, involved in the activation of Th1 and Th17-mediated immune response, respectively. The better understanding of pathogenetic mechanisms of IBD has allowed the identification of processes or pathways that could be targeted by new drugs. They can be summarized as follows: (1) the egress of activated lymphocytes from secondary lymphoid tissue; (2) the tissue homing of lymphocytes; (3) the activity of specific pro-inflammatory or anti-inflammatory cytokines; and (4) the JAK/STAT signaling pathway, which is common to several cytokines (**Figure 1**). S1P receptor agonists represent the first group, which includes three orally administered small molecules on clinical development: ozanimod, amiselimod and etrasimod. Among them, ozanimod is the most advanced in clinical development for UC, and already published phase 2 clinical data seem very promising. Treatment with ozanimod, in fact, is effective for inducing clinical remission already at week 8 and maintaining up to week 32, in a good percentage of patients. With regard of safety, for its selectivity for S1P_1_ and S1P_5_ receptors, ozanimod seems having a better profile, compared to its predecessor fingolimod, in terms of cardiovascular effects, pulmonary disorders and elevation of serum liver enzymes (Juif et al., 2016). Moving forward on the second group, the inhibition of lymphocytes adhesion to endothelial cells, mediated by the interaction between α_4_β_7_ integrin and MAdCAM-1, is the key target for pharmacological interventions. As far as anti-integrin drugs are concerned, three new molecules have been developed: abrilumab, a monoclonal antibody sharing the same mechanism of action with vedolizumab, but administered s.c., AMJ300 which is an oral, small molecule binding α4 subunit, and etrolizumab, a monoclonal antibody administered s.c., direct against β_7_ subunit. PF-00547659 is the first anti MAdCAM-1 in clinical development for IBD. Overall, the efficacy data of phase 2 studies with these molecules confirm the already observed late onset of action of this class, especially for CD (abrilumab and PF-00547659 failed induction primary endpoints), potentially related to transmural features of this disease (Sandborn et al., 2013). Conversely, phase 2 study for etrolizumab in UC showed encouraging results (Vermeire et al., 2014). Among pro-inflammatory cytokines, the selective inhibition of IL-23 and IL-6 by monoclonal antibodies (risankizumab and brazikumab i.v., and PF-04236921 s.c., respectively), showed short-term clinical benefit in patients with refractory CD patients in phase 2 studies (long-term data are still awaited). Within this group of new targets, the potential development of phosphodiesterase 4 selective inhibitors deserves a mention. Two drugs of this class already have authorized therapeutic uses: roflumilast for chronic obstructive pulmonary disease, and apremilast for psoriatic arthritis and chronic plaque psoriasis. Apremilast is on phase II clinical development for UC (NCT02289417). The increased intracellular cAMP levels induced by apremilast in immune cells trigger signaling pathways that produce anti-inflammatory effects (Keating, 2017). In particular, apremilast inhibits the secretion of TNF-α from human peripheral blood (Schafer et al., 2014) and colonic lamina propria (Gordon et al., 2009) mononuclear cells, and selected Th1, Th2, and Th17 cytokines, including IL-2, IL-17, TNF-α, and IFN-γ, from human T cells (Schafer et al., 2014). A novel pharmacological approach for IBD is interfering with transduction of cytokines-mediated signal, through the inhibition of the activity of JAK family members. Tofacitinib, an oral small molecule, already on the market for RA, will be reasonably soon approved for treatment of UC. The results of phase 3 OCTAVE program, in fact, have prompted great interest on this molecule, showing rapid onset of action, durable efficacy, and a favorable safety profile (Feagan et al., 2016c; Sandborn et al., 2017d). Another appealing compound is mongersen, an oligonucleotide designed to reduce the cytosolic levels of the inhibitory Smad, Smad7, which produces down-regulation of receptors for TGF-β, and thereby restore the immunosuppressive properties of TGF-β, which has shown, in phase 2 study, surprising results in terms of clinical benefit after only 2 weeks of treatment (Monteleone et al., 2015). Looking forward to the next few years, healthcare providers will probably have access to some of these compounds in clinical practice. The big challenge will be the correct positioning of each one in treatment algorithms for IBD, according to pharmacological properties, efficacy in specific population and safety.

## Author Contributions

Conception of work: DC; Design of work: DC, DP, and AA; Drafting of manuscript: DC and DP; Critical revision of manuscript: DC, DP, and AA; Final approval of work: DC, DP, and AA; Agreement to be accountable for all aspects of presented work: DC, DP, and AA.

## Conflict of Interest Statement

DP: Lecture fee(s) from AbbVie, MSD, and Takeda. AA: Consultant and lecture fee(s) from AbbVie, AstraZeneca, Chiesi, Ferring, Hospira, Lilly, MSD, Mundipharma, Otsuka, Sofar, Takeda, Zambon; grant for research from MSD. The other author declares that the research was conducted in the absence of any commercial or financial relationships that could be construed as a potential conflict of interest. The reviewer AR and handling Editor declared their shared affiliation, and the handling Editor states that the process nevertheless met the standards of a fair and objective review.

## Abbreviations

AE: adverse event
AUC: area under the plasma concentration-time curve
CD: Crohn’s disease
CDAI: Crohn’s disease activity index
C_max_: maximal plasma concentration
GM-CSF: granulocyte-macrophage colony-stimulating factor
IBD: inflammatory bowel disease
IFN: interferon
IL: interleukin
i.v.: intravenous
JAK: Janus kinase
MAdCAM-1: Mucosal Vascular Addressin Cell Adhesion Molecule 1
MH: mucosal healing
MS: multiple sclerosis
NK: natural killer
OLE: open-label extension
p.o: *per os*
PML: progressive multifocal leukoencephalopathy
RA: rheumatoid arthritis
STAT: Signal Transducer and Activator of Transcription
S1P: sphingosine 1-phosphate
s.c.: subcutaneous
TGF: transforming growth factor
Th: T helper
TNF-α: tumor necrosis factor-α
TYK2: tyrosine kinase 2
UC: ulcerative colitis
VCAM-1: Vascular Cell Adhesion Molecule-1
